# Identification and Characterization of *C-Mos* in Pearl Mussel *Hyriopsis cumingii* and Its Role in Gonadal Development

**DOI:** 10.3390/biom13060931

**Published:** 2023-06-01

**Authors:** Zongyu Liu, Xin Jin, Yulin Miao, Ping Wang, Yang Gu, Xiaozhao Shangguan, Lijing Chen, Guiling Wang

**Affiliations:** 1 Key Laboratory of Freshwater Aquatic Genetic Resources, Ministry of Agriculture and Rural Affairs, Shanghai Ocean University, Shanghai 201306, China; 2 National Demonstration Center for Experimental Fisheries Science Education, Shanghai Ocean University, Shanghai 201306, China; 3 Shanghai Engineering Research Center of Aquaculture, Shanghai 201306, China; 4 Shanghai Vocational College of Agriculture and Forestry, Shanghai 201699, China

**Keywords:** *C-Mos*, gonadal development, oocyte maturation, RNAi, *Hyriopsis cumingii*

## Abstract

*C-Mos*, a proto-oncogene, regulates oocyte maturation by activating the classical MAPK pathway in cells. To examine the function of *C-Mos* in *Hyriopsis cumingii*, *C-Mos* was identified in this study. The full-length cDNA of *C-Mos* was 2213 bp, including 144 bp in the 5′ UTR, 923 bp in 3′ the UTR, and 1146 bp in the open reading frame (ORF) region. During early gonad development, the expression of *C-Mos* from 4 to 6 months of age in *H. cumingii* was significantly higher than that in other months, with the highest expression in 6-month-old *H. cumingii*, suggesting that *C-Mos* may be involved in early gonadal development in *H. cumingii*. Clear hybridization signals were found by in situ hybridization in the oocytes, oocyte nucleus and oogonium, and a small number of hybridization signals were found in the follicular wall of the male gonads. In addition, the *C-Mos* RNA interference (RNAi) assay results showed that the knockdown of *C-Mos* caused a down-regulation of *ERK* and *P90rsk*. In summary, these results indicate that *C-Mos* has a crucial part to play in gonadal development in *H. cumingii*.

## 1. Introduction

The MAPK (mitogen-activated protein kinase) pathway is widely expressed in animals, regulates varieties fundamental cellular events and is closely related to cell proliferation and differentiation [1]. The MAPK pathway can be divided into four cascade pathways: classical MAPK, p38-MAPK, JNK-MAPK, and ERK5-MAPK. Each cascade comprises three to five layers, depending on the substrate being acted upon [2]. Three of these layers (Mapkkk, Mapkk, and Mapk) have been identified as essential layers. Upon extracellular stimulation and activation by various internal cellular processes, single or multiple kinases within their layers can phosphorylate and activate downstream signalling molecules to initiate the cascade. Much of the current research on the MAPK pathway in reproduction has focused on the classical MAPK pathway and the P38-MAPK pathway, which have a significant impact on promoting gonadal development and regulating oocyte maturation [3,4,5]. In *Cladonema pacificum*, dephosphorylation of MAPkinase leads to the release of eggs from the stagnant state. The same result was obtained after MAP kinase kinase (MEK) inhibition using U0126 [6]. In *Urechis caupo*, active MAPK appears to be required for normal meiotic divisions and suppressing the paternal centrosome until after the egg completes meiosis [7]. Upon receipt of a stimulus, the upstream scaffold protein organises the various members of the pathway, delivers the signal precisely to the nucleus, and activates various transcription factors [8]. In mice, the MAPK pathway is implicated in sex determination [9] and modulates *Sox9* expression to influence testicular development [10]. In *Caenorhabditis elegans*, the MAPK pathway is essential for the development of oocyte [11].

*C-Mos*, a proto-oncogene, is homologous to Moloney’s murine sarcoma virus oncogene. It is specifically expressed in the gonads, and is able to activate the MAPK pathway in oocytes of numerous species, except for *Caenorhabditis elegans*, which do not possess *C-Mos* [12]. Progesterone secreted from peripheral follicle cells binds to membrane receptors in the oocyte to initiate egg maturation, inducing a downward trend in intracellular cAMP levels, which inactivates PKA and stimulates the translation of *C-Mos*. Cyclin-B1 (CCNB1) and Cyclin-dependent Kinase 1 (cdk1) together form the maturation-promoting factor (MPF), which plays a key role in meiosis entry. Works in various species have revealed that MPF is strongly connected to the MAPK pathway [13,14,15]. In the *Xenopus*, *C-Mos* activates MPF [16] and the injection of *C-Mos* RNA into oocytes can successfully activate MPF and cause oocytes germinal vesicle breakdown (GVBD), even if the oocytes are not stimulated by progesterone [17]. *C-Mos* shows significant spatiotemporal expression in mouse testes and is suggested to be involved in spermatogenesis [18]. Oocyte development in vivo is not continuous, and is influenced by many factors during the process [19,20,21]. Oogenic cells receive hormonal stimulation and are induced to enter meiosis by GVBD [22], followed by oocyte arrest in the MII phase awaiting fertilization in vertebrates and mostly in the MI phase in molluscs [23]. Cell cycle arrest in eggs is found in amphibian *Rana pipens* oocytes and is regulated by a cytostatic factor (CSF) closely related to the MAPK pathway [24]. *C-Mos* was first identified as part of the CSF in the *Xenopus tropicalis* in 1989 [25]. Several studies have pointed to an important function for the *C-Mos-ERK* (extracellular signal-associated kinase) pathway in oocyte arrest [26,27].

A rich variety of sex types have been identified in shellfish, such as hermaphroditism, male prematurity and sexual reversal [28,29], and the high plasticity of the gonads indicates that shellfish are ideal species for studying sex systems. *Hyriopsis cumingii*, a freshwater bivalve mollusc, is an important pearl-bearing mussel endemic to China, with a high pearl-producing performance, making it a highly desirable economic shellfish for aquaculture. It has been shown that male *H. cumingii* have a higher pearl production performance than females [30]. However, no mechanism for sex determination in *H. cumingii* has been identified, and no heteromorphic chromosomes have been found, so the genes involved in regulating sex and gametogenesis in *H. cumingii* are the focus of research.

In this study, *C-Mos* was identified. The expression characteristics of *C-Mos* in different tissues and at different ages was analysed. We also characterised the part played by *C-Mos* in the gonads by in situ hybridization and RNA interference (RNAi). The results indicate an importance of *C-Mos* for *H. cumingii* gonadal development and oocyte maturation.

## 2. Materials and Methods

### 2.1. Experimental Material and Sample Collection

Juvenile and sexually mature *H. cumingii* used in this study were taken from the Wuyi experimental farm in Jinhua, Zhejiang Province. After arrival at the laboratory, the *H. cumingii* were divided into tanks and kept at a water temperature of 26 °C ± 2 °C for 8 days. The tissues (liver, gill, adductor, mantle, foot and gonad) were collected from 36-month-old *H. cumingii* (three females and three males). Gonadal tissues with three biological replicates of each sample were collected from mussels between 1 and 8 months of age, and between 1 and 3 years old. All samples for RNA extraction were taken and rapidly frozen in a tube using liquid nitrogen before being stored at −80 °C. Then, 5 mm blocks of gonadal tissue were extracted from 36-month-old *H. cumingii* to be fixed in paraformaldehyde and used to make paraffin sections.

### 2.2. Obtaining RNA and cDNA

Total tissue RNA was obtained with the Trizol method, 1% agarose gel electrophoresis was used to detect whether the RNA was degraded and nanodrop2000 (Thermo Fisher Scientific, Waltham, MA, USA) was used to measure the RNA concentration and the 260/280. The RNA was reverse transcribed to cDNA in vitro using the PrimeScript RT Reagent Kit with gDNA Eraser kit (TaKaRa, Dalian, Japan) according to the manufacturer’s instructions and then diluted fivefold and stored at −40 °C for PCR and quantitative real-time PCR (qRT-PCR).

### 2.3. The Full-Length Cloning of C-Mos and Its Sequence Analysis

Partial sequence information for *C-Mos* was obtained from the transcriptome library, and the 3′ race primers (Table 1) were designed according to this sequence. The SMARTer RACE 5′/3′ Kit (Clontech, Mountainview, CA, USA) was used for 3′ RACE cloning of *C-Mos* according to the instructions. The PCR product was cloned to the pmd19-T vector. The plasmid was transfected into receptor *E. coli* dh5α and amplified with AMP^+^ liquid medium at 37 °C and 200 rmp for 6 h. Then, 100 µL of the amplified broth was evenly applied to blue and white spot medium and cultured. The white spots were selected for sequencing.

NCBI ORFfinder (https://www.ncbi.nlm.nih.gov/orffinder/) (accessed on 15 March 2023) was used to predict the ORF of *C-Mos*; analysis of basic physicochemical properties of proteins was undertaken using the ProtParam tool (https://web.expasy.org/aprotparam/) (accessed on 15 March 2023); SMART was used to predict the kind of protein domain and its position on the sequence (http://smart.embl-heidelberg.de/smart) (accessed on 15 March 2023); tertiary structures were predicted using the SWISS-MODEL program (https://swissmodel.expasy.org/) (accessed on 15 March 2023); the different amino acid sequences were analysed for homology using ClustalW in BioEdit 7.0.9.0 (BioEdit, Manchester, UK); and phylogenetic trees were constructed using Neighbour Joining (NJ) and repeated 1000 times to confirm confidence values among species in the Mega 11.0 program (Supplementary Table S1).

### 2.4. Quantitative Real-Time PCR

Biological triplicates cDNAs were mixed together and used for qRT-PCR. qRT-PCR was performed using the Bio-Rad real-time CFX96 PCR system (Bio-Rad, Hercules, CA, USA). The *EFl-α* was used as a reference gene. The qRT-PCR procedure is based on techniques from Gu [31]. Both the target and reference genes were analyzed in triplicate. The 2^−∆∆CT^ method of calculating relative expressions was used and Prism 8.0 (GraphPad, San Diego, CA, USA) was used to produce the plots. The relative expression of the other groups was determined by using the expression of the control group as 1 in each graph. In cases where a control group was not available, the lowest expression value among all data sets was used as the reference value of 1.

### 2.5. Preparation of Paraffin Sections and In Situ Hybridization (ISH)

After sampling, the tissue was immediately placed into a 1.5 mL Eppendorf tube containing 1 mL of 4% paraformaldehyde. The tissue was soaked at 4 °C for 2 h prior to starting the dehydration process. The dehydration steps included soaking the tissue in 70% ethanol for 2 h, 80% ethanol for 30 min, 90% ethanol for 15 min, 95% ethanol for 10 min, and absolute ethanol twice for 10 min and 5 min, respectively. Next, the tissue was rendered transparent through immersion in a solution of xylene and absolute ethanol (1:1) for 20 min, followed by two immersions in xylene for 5 min and 2 min, respectively. After this, the tissue was embedded in a tissue embedding cassette and kept in a solution of paraffin and xylene (1:1) at 65 °C for 1 h, followed by another 1 h incubation in paraffin solution at 65 °C and a final incubation for 30 min in paraffin solution at 65 °C. Finally, the tissue was allowed to cool and then sectioned.

The probe template was synthesised by adding a T7 promoter to the 5′ end of a gene specific reverse primer (Table 1), amplified by PCR, purified, and then used for in vitro transcription. It was reverse transcribed as an antisense probe in vitro using the T7 High Efficiency Transcription Kit (Transgen, Beijing, China) and DIG RNA Labeling Mix. The probes were purified and then stored at −80 °C. After removal of paraffin, in situ hybridization was performed according to the instructions of the Enhanced Sensitive ISH Assay Kit II (Boster, Wuhan, China), catalog number MK1032. The thickness of the paraffin sections used for in situ hybridization was 6 µm. The main steps in ISH were as follows: the tissue was digested for 60 s using freshly diluted pepsin (two drops of pepsin per 1 mL of 3% citric acid), then washed with 0.5 M PBS at room temperature, and the procedure was repeated three times. A wet cassette was arranged using 20% glycerol moistened cotton balls, and the next steps were carried out in the wet cassette. Prehybridization solution was added and it was incubated for 3 h at 37 °C. The sections of the experimental group were then added with a concentration of 1 ng/µL of the probe, and the control group was not added. The coverslip was put in place on the section and it was incubated overnight (16 h) at 37 °C. Next, the tissue was washed at 37 °C with 2 × SSC for 5 min, repeated once. It was washed at 37 °C with 0.5 × SSC for 15 min, then at 37 °C with 0.2 × SSC for 15 min. The closure solution was added dropwise at 37 °C for 30 min. Biotinylated murine anti-digoxin was added dropwise, and it was incubated at 37 °C for 1 h. The tissue was washed with 0.5 M PBS at 37 °C for 5 min, repeated four times. SABC-AP was added dropwise, at 37 degrees for 30 min. Then, 0.01 M TBS was washed at 37 °C for 5 min, repeated four times. Finally, BCIP/NBT was added dropwise for colour development at 37 °C for 5 min, rinsed well with distilled water, and the film sealed. The tissue after in situ hybridization was observed using the microscope (Leica DM 2500, Leica, Wetzlar, Germay) photographed with a Leica DFC450, and the experimental results observed under a visible light source.

### 2.6. RNAi Assay

Three pairs of double-stranded RNA (dsRNA) primers G1, G2, and G3, were designed using primer 5.0 in the ORF region of the *C-Mos* (Table 1). The amplified regions of these primers did not contain those of the qRT-PCR primers for *C-Mos*. The length of the G1 interference chain was 518 bp, the length of the G2 interference chain was 437 bp and the length of the G3 interference chain was 470 bp. The green fluorescent protein (GFP) sequence, which had no similarity to the *C-Mos* of the *H. cumingii*, was selected as the interference chain for the negative control group (NG). The synthesis of dsRNA was based on the method of Wang [32]. dsRNA concentration was adjusted to 400 ng/µL working concentration and stored at −80 °C.

12-month-old *H. cumingii* with a shell length of 7 cm was selected for RNAi experiments. The gonads of *H. cumingii* were aspirated using a syringe, then applied to slides and finally observed with a microscope (Leica DM 2500) to pre-identify males and females. The mussels were randomly assigned to four groups (G1 group, G2 group, G3 group and NG group), each group having 30 mussels. Each mussel was injected with 50 µL of interfering chain at a concentration of 400 ng/µL using a 100 µL syringe; the location of the injection was the adductor. At the time of sampling, 10 gonadal tissue samples were collected from each group at 12, 24 and 48 h post-injection and used to make paraffin sections and for RNA extraction. After the paraffin sections had been stained with haematoxylin and eosin to identify the sex of the *H. cumingii*, three female gonads were randomly selected from each group at each time point to extract RNA, reverse transcribe it into cDNA and then perform qRT-PCR. The expression levels of *C-Mos*, *ERK* and *P90rsk* (90-kDa ribosomal S6 protein kinase) were measured using qRT-PCR. The primers are listed in Table 1.

### 2.7. Statistical Analysis

An independent samples *t*-test and one-way analysis of variance ANOVA was conducted using SPSS 26 software (IBM, Chicago, IL, USA) to verify whether there was a significant difference between the data sets. *p* < 0.05 was regarded as a significant difference, indicated by an asterisk. *p* < 0.01 was regarded as a highly significant difference, indicated by two asterisks. All data obtained were plotted as mean ± standard deviation.

## 3. Results

### 3.1. Full-Length and Sequence Characteristics of the cDNA of C-Mos

**Figure 1:**
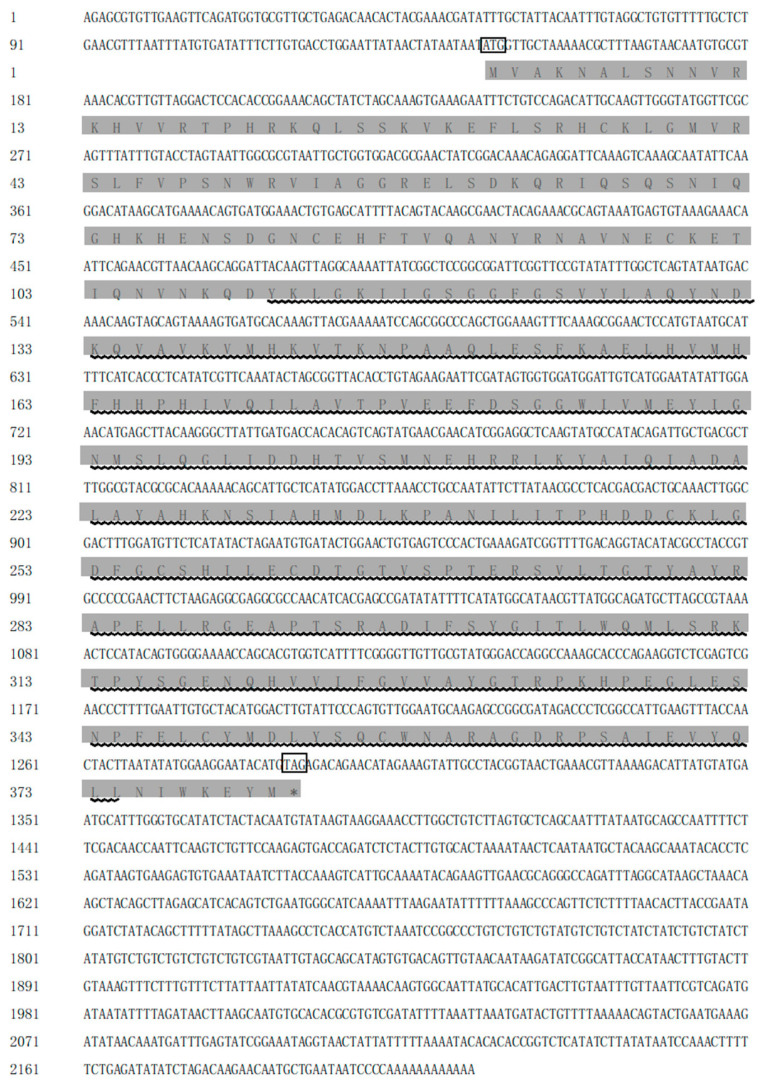
*C-Mos* nucleotide sequence and amino acid sequence of *H. cumingii*. ATG is the start codon and TGA is the stop codon, both are marked with boxes. The shaded area is the amino acid sequence corresponding to the open reading frame, and the wavy part is the S-TKC structural domain (111-374 aa).

**Figure 2:**
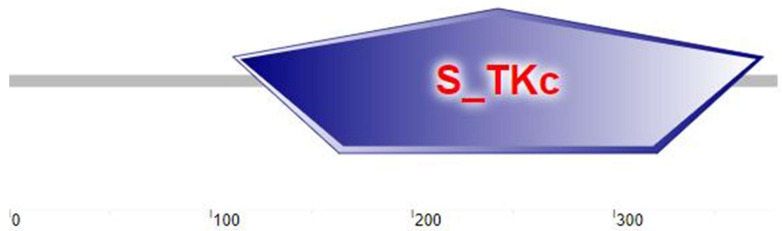
The result of the secondary structure prediction of the C-Mos protein in *H. cumingii* indicates that the C-Mos protein has an S-TKC structural domain (111-374 aa). The *C-Mos* cDNA sequence encoding 381 amino acids. The number represents the position of the C-Mos protein amino acid.

**Figure 3:**
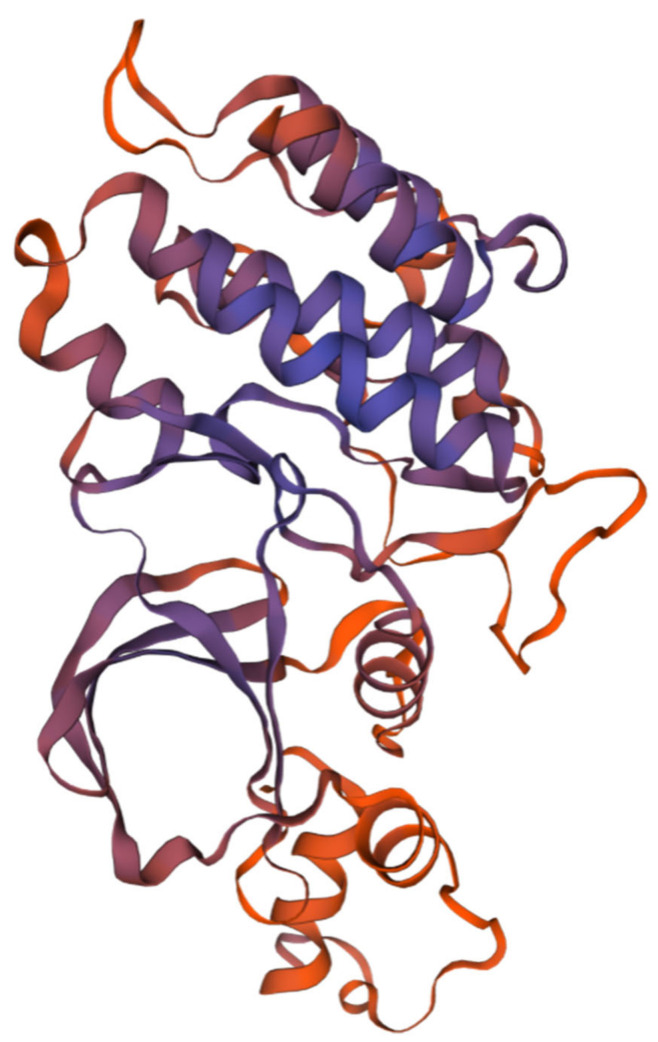
The tertiary structure of the C-Mos protein predicted with SWISS-MODEL in *H. cumingii,* the darker the colour, the higher the level of confidence.

This study clones the *C-Mos* cDNA sequence from *H. cumingii*, with a 5′ UTR length of 144 bp, a 3′ UTR length of 923 bp and an ORF region length of 1146 bp, encoding 381 amino acids (Figure 1), the GenBank accession number is OQ161186. The calculated molecular mass of the C-Mos protein is predicted to be 43.07 kDa, with an isoelectric point of 8.63 and a hydrophilicity coefficient of −0.417. According to the analysis, it is a hydrophilic protein. However, it is assumed that the C-Mos protein is an intracellular protein as it possesses neither a transmembrane structure nor signal peptide properties. The C-Mos protein has a serine/threonine protein kinase catalytic (S-TKC) structural domain (111-374 aa), which possesses ATP-binding capacity and serine/threonine protein kinase activity (Figure 2). The protein tertiary structure of the C-Mos was predicted using SWISS-MODEL based on the 1k9a. 2. A template, which shared 31.13% identity with the C-Mos protein. The alpha helix accounted for 27%, the beta strand for 15% and irregular curl for 23% (Figure 3).

**Figure 4:**
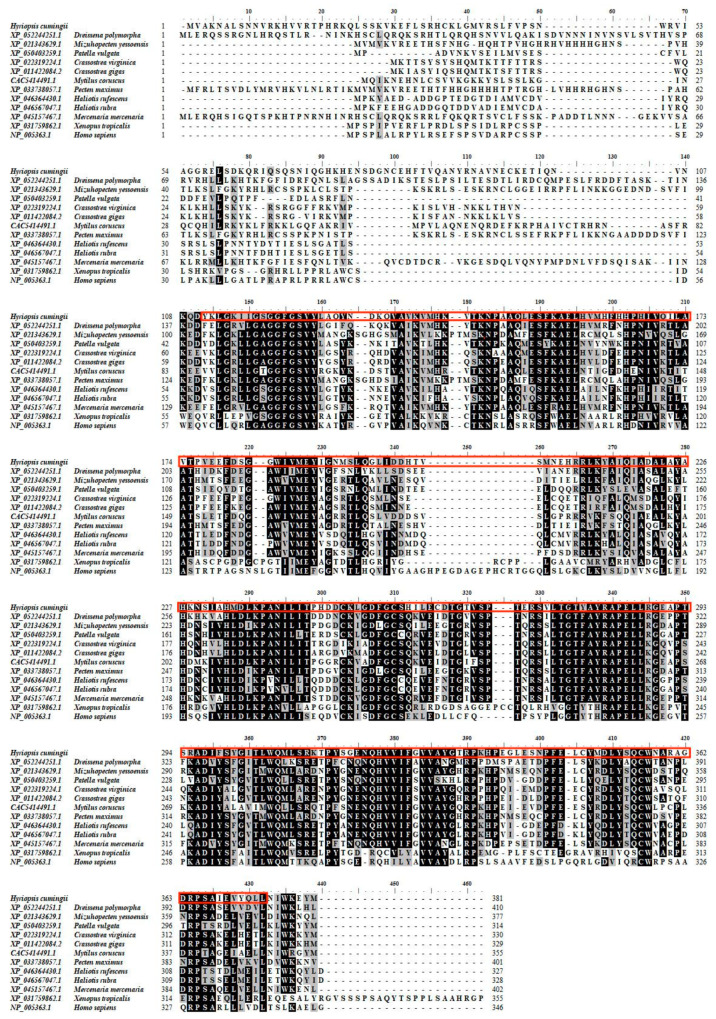
Multiple alignment analysis of C-Mos protein between *H. cumingii* and other species. The amino acid sequence highlighted in the red box is the S-TKC structural domain of the C-Mos protein of the *H. cumingii*. The different colours in the image indicate the degree of consistency. The heavier the colour, the greater the degree of agreement.

**Figure 5:**
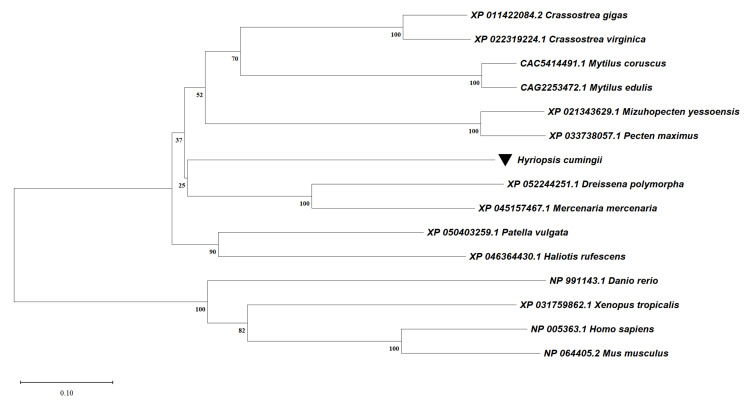
NJ Phylogenetic tree of C-Mos protein of *H. cumingii* and other species. The length of the distance scale in the bottom left corner represents an evolutionary distance of 0.1, with larger distances representing higher levels of gene variation compared to the original ancestor.

Sequence alignment between species was analysed using BioEdit (Figure 4). The C-Mos protein is conserved between the *H. cumingii* and other species. The highest homology was with *Dreissena polymorpha* (XP_052244251.1), at 62.23%. Homology with *Mizuhopecten yessoensis* (XP_021343629.1) and *Patella vulgata* (XP_050403259.1) was 60.07% and 59.78%. The high level of resemblance (up to 67.80%) between the S-TKC structural domain of the C-Mos protein of many organisms suggests evolutionary and structural conservation, thus predicting the functional conservation of C-Mos in protein kinases. The results of the phylogenetic tree show that *H. cumingii* are clustered on one branch with other bivalves. The vertebrates are also clustered on one branch. Among the bivalves, the evolutionary distance of *H. cumingii* is medium (Figure 5).

### 3.2. Expression Analysis of C-Mos in Different Tissues of the H. cumingii

**Figure 6:**
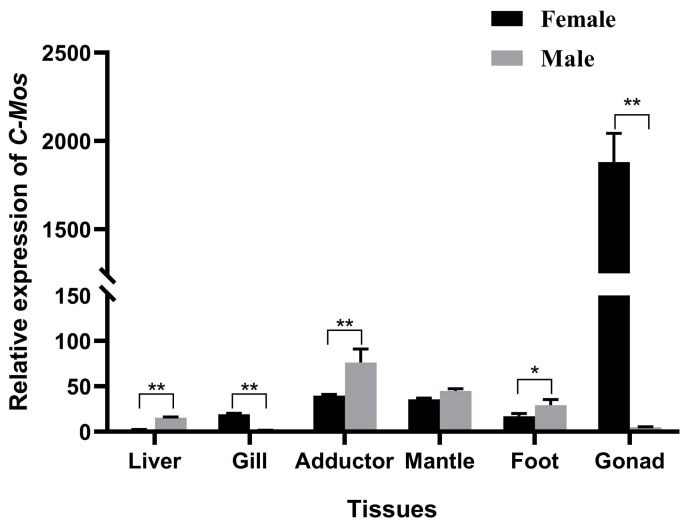
Relative expression of *C-Mos* genes in different tissues of the *H. cumingii.* The expression of *C-Mos* in the gills of male *H. cumingii* was set as 1 to calculate the relative expression of *C-Mos* in other tissues. * indicate a significant difference between the data and ** indicate a highly significant difference between the data.

The expression of *C-Mos* was examined by qRT-PCR across different tissues of *H. cumingii* (Figure 6). There were significant differences in *C-Mos* expression among *H. cumingii* tissues (*p* < 0.05). In males, *C-Mos* was expressed at the highest level in the adductor. In females, *C-Mos* was most highly expressed in the female gonads and significantly higher than in the male gonads and all other tissues (*p* < 0.01).

### 3.3. Analysis of C-Mos Expression at Different Ages in the H. cumingii

**Figure 7:**
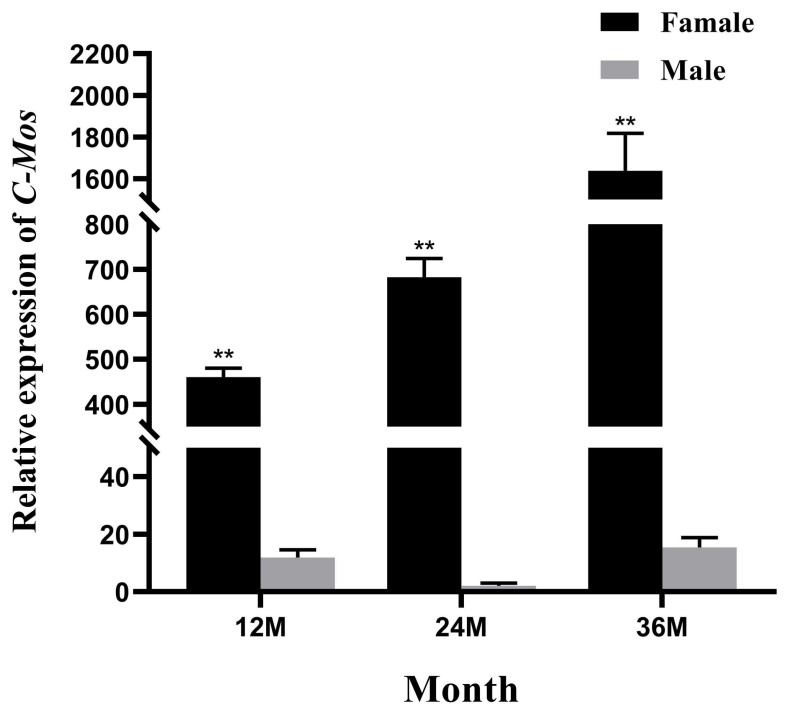
Relative expression of *C-Mos* in gonadal tissues of *H. cumingii* at different ages. The expression of *C-Mos* in the 24-month-old male *H. cumingii* gonads was set as 1 to calculate the relative expression of *C-Mos* in the gonads of other conditions. ** indicate a highly significant difference between the data.

The expression of *C-Mos* in the gonads of *H. cumingii* at different ages was detected using qRT-PCR. The expression of *C-Mos* showed a gradual increase in 12 to 36-year-old *H. cumingii*, with the highest expression at age three. It was higher in females than in males at 12, 24 and 36 months old (*p* < 0.01), and almost absent in males (Figure 7).

### 3.4. Analysis of C-Mos Relative Expression during the Juvenile Period of the H. cumingii

**Figure 8:**
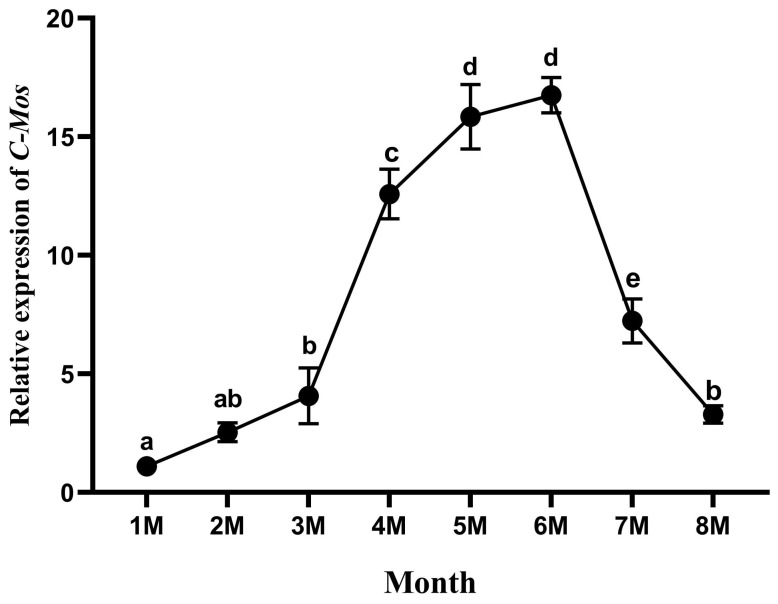
Relative expression of *C-Mos* in gonadal tissues of *H. cumingii* at different months of age. The expression of *C-Mos* in the one-month-old *H. cumingii* was set as 1 to calculate the relative expression of *C-Mos* in the gonads of other conditions. Different letters (a, b, c, d, e) indicate significant differences between the data (*p* < 0.05), and the same letters indicate no significant difference between the data (*p* < 0.05).

The expression of *C-Mos* in the gonads of *H. cumingii* at different months of age was detected using qRT-PCR. In the early gonadal development of juvenile *H. cumingii* (1–8 months old), the expression of *C-Mos* showed an initial increase followed by a decrease over time. At 4, 5 and 6 months of age, a marked increase in the expression of *C-Mos* was detected (*p* < 0.05), when the gonads are differentiated in the *H. cumingii*, reaching a peak in 6 months of age (Figure 8).

### 3.5. Localization of the C-Mos

**Figure 9:**
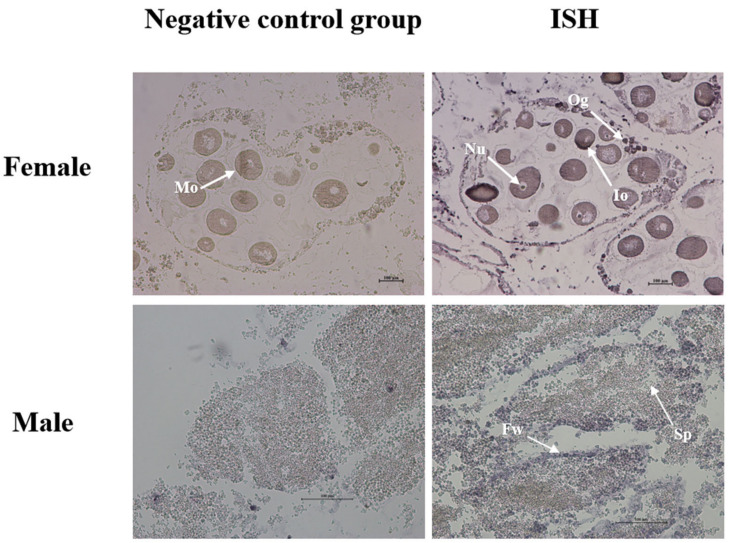
In situ hybridization of *C-Mos* in female and male gonads. The hybridization signal is blue–purple. Io: immature oocyte, Mo: mature oocyte, Og: oogonium, Nu: nucleus, Sp: sperm, Fw: follicular wall.

The sections were observed and the localization of *C-Mos* in the gonad was determined after in situ hybridization, with the signal stronger in the female gonads than in the male gonads, whereas no signal was found in either negative control (Figure 9). Clear hybridization signals were found by in situ hybridization in the oocytes and the nucleus of the oocyte and oogonium, and a small number of hybridization signals were found in the follicular wall of the male gonads; no signals were found in other areas.

### 3.6. RNAi of C-Mos

**Figure 10:**
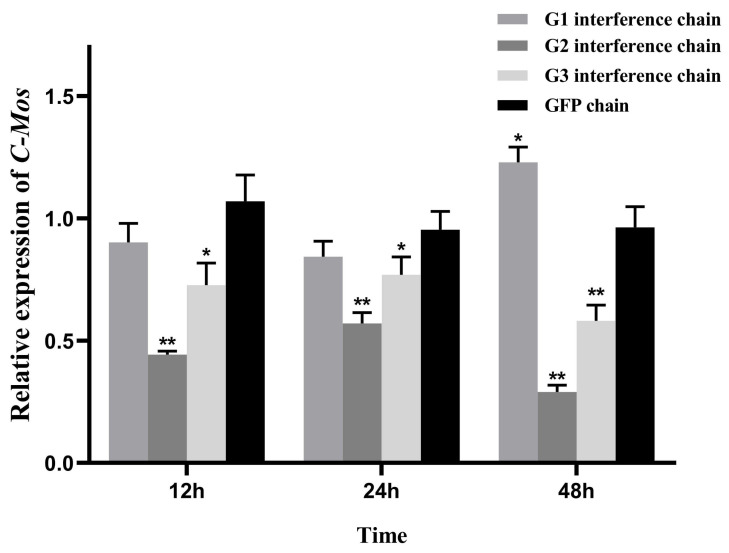
Relative expression of *C-Mos* in female gonads of *H. cumingii* following RNAi experiments. The expression of *C-Mos* in the gonads of *H. cumingii* in the NG group was set as 1 to calculate the relative expression of *C-Mos* in the gonads of other conditions. * indicate that the data is significantly different from the control group (*p* < 0.05), and ** indicate that the data is highly significantly different from the control group (*p* < 0.01).

Interference chains G1, G2, G3 and GFP were injected into the adductor of each mussel of groups G1, G2, G3 and NG, respectively. Relative expression of *C-Mos* in female gonads of *H. cumingii* following RNAi experiments was detected using qRT-PCR. In comparison with the NG group, the G2 and the G3 groups showed a significant interference effect. According to the previous experimental experience, not every interference chain can produce interference effect. In this experiment, the G1 interference chain has no interference effect (Figure 10). The interference rates in the G2 group were 58.59%, 40.19% and 69.89% at 12 h, 24 h, and 48 h, respectively. After 12 h, 24 h, and 48 h, the G3 group experienced interference rates of 32.07%, 19.31%, and 39.72% respectively. The G2 group was chosen to explore the function of *C-Mos* based on the effect of interference.

### 3.7. Expression Analysis of ERK and P90rsk after RNAi

**Figure 11:**
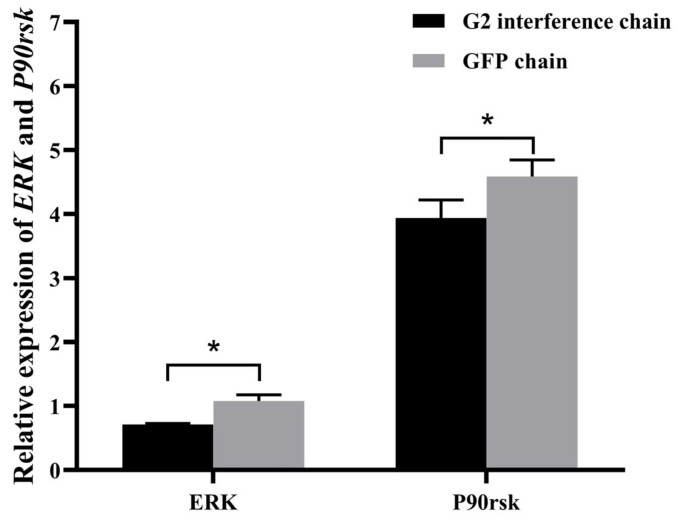
Relative expression of *ERK* and *P90rsk* in female gonads of *H. cumingii* following RNAi experiments. The expression of *ERK* in the gonads of *H. cumingii* in the NG group was set as 1 to calculate the relative expression of *ERK* and *P90rsk* in the gonads of other conditions. * indicates a significant difference between the data.

To assess the ability of *C-Mos* to function in *H. cumingii*, the expression levels of *ERK* and *P90rsk* in female gonads of the G2 group and the NG group were probed 48 h after injection (Figure 11). Compared to the NG group, the interference rate of *ERK* at 48 h was 31.83% and the interference rate of *P90rsk* at 48 h was 14.12%.

## 4. Discussion

The *C-Mos* gene was successfully cloned in this experiment. Its full length of 2213 bp encoded 381 amino acids (Figure 1), and a protein structure prediction analysis revealed that it contained an S-TKC structural domain (111-374 aa) (Figure 2) with conserved catalytic activity. *C-Mos* is a serine/threonine kinase whose main role is to regulate intracellular activity by phosphorylating or dephosphorylating substrates. An essential piece of the catalytic structural domain is 253D, a highly conserved residue positioned in the central region. A glycine-rich segment of the S-TKC structural domain is thought to participate in the ATP binding process. The S-TKC structural domain is conserved among various species [33,34], and participates in reproductive processes in a wide range of species. In *Atrina pectinata*, *tssk* plays a role in spermatogenesis [34] and the *CDK* of *Dendrobium candidum* has a function in regulating the embryonic cell cycle [35]. There was a high conservation of *C-Mos* across species, which implies that in vertebrates and molluscs it may share similar functions. The results of the phylogenetic tree showed that *H. cumingii* are clustered on one branch with other bivalves. The vertebrates are also clustered on one branch. This shows that *H. cumingii* has a high homology with bivalves and a low homology with vertebrates. Among the bivalves, the evolutionary distance of *H. cumingii* was medium, indicating that the evolutionary extent of the C-Mos protein in the mussel is intermediate in bivalves.

In the present study, the *C-Mos* gene was expressed in the foot, liver, gill, mantle, gonad, and adductor of the *H. cumingii*, with a high expression specifically in the female gonads and highly significant differences compared to other tissues (*p* < 0.01). This reveals a possible function in the female gonads. In mice, *C-Mos* was expressed at low levels in kidney, brain, mammary gland, and placenta, and at high levels in gonads [36], similar to the present results. The same result was also found in *Clytia* [37]. Past studies have found that the MAPK cascade is a participant that helps to control the cell cycle of mammalian oocytes, particularly spindle assembly and microtubule organization during mammalian oocyte meiosis [38], controlling the oocyte cycle and promoting oocyte maturation [39]. The precocious translation of *C-Mos* can also facilitate the meiotic maturation of oocytes [40]. The expression of *C-Mos* showed a gradual increase at 1, 2 and 3 years of age, with the highest expression at age 3 and with a highly significant difference in expression between males and females (*p* < 0.01). The gonads of the *H. cumingii* complete their differentiation at the age of one year and become sexually mature at 3 years old. This pattern of expression of *C-Mos* in *H. cumingii* suggests that it might have an important contribution in the maturation to the gonads. To examine the influence of *C-Mos* in the gonads of *H. cumingii* further, we examined the expression of *C-Mos* in the gonads of juvenile *H. cumingii*. The gonads of *H. cumingii* in this study showed a gradual increase in *C-Mos* expression from 1 to 6 months of age, reaching a maximum in 6 months of age and gradually decreasing from 7 to 8 months of age. Primordial germ cells (PGCs) appeared in the tissues of *H. cumingii* at 4 months of age [41]. *C-Mos* may be implicated in the *H. cumingii* PGCs’ proliferation due to its high expression between the ages of 4 and 6 months. In *Rana esculenta*, the MAPK pathway is able to regulate spermatogonia proliferation [42]. In zebrafish, the MAPK pathway can promote the proliferation of PGCs [43]. The results suggest that the *C-Mos* may be involved in the proliferation of PGCs in the *H. cumingii* through the regulation of the MAPK pathway.

Oocyte maturation goes through the physiological process of GVBD. This is accompanied by chromatin aggregation, nuclear membrane rupture and the inability to carry out physiological functions such as transcription and shearing. Therefore, residual maternal transcripts in cells can drive the oocyte maturation process. [44]. The translation timing of maternal transcripts of *C-Mos*, which accumulated gradually during the GV phase, are controlled by the combined action of factors such as *Gld2* (germline development 2) and PARN (poly(A) ribonuclease) [45], which allows it to be expressed during meiosis. Our study clearly detected hybridization signals of *C-Mos* in oocytes, oocyte nucleus, and oogonium. A small number of hybridization signals were found in the follicular wall of the male gonads by in situ hybridization, suggesting that *C-Mos* may be involved in the oocyte development process of *H. cumingii*. In vertebrates, at MII stage, *C-Mos* expression in oocytes reaches its peak [16]. In zebrafish, transcripts of *C-Mos* were found to be detectable throughout the oocyte during early oogenesis [46].

In the absence of C-Mos proteins, sea star oocytes enter a repetitive embryonic mitotic cycle after meiosis I, and its rein-statement restores meiosis II and subsequent cell cycle arrest [47]. Here we knock down *C-Mos* by RNAi to examine the possible function of *C-Mos* in gonadal development. In this experiment, the interfering chain G2 group had the best interference effect at 48 h post-injection, with a 69.89% *C-Mos* gene interference rate compared to the NG group. Additionally, we measured both *ERK* and *P90rsk* expression, the genes of the MAPK pathway which mediates oocyte arrest, in order to better understand the function of *C-Mos*. *ERK* is a key kinase of the classical MAPK pathway and plays a major part in mammalian reproduction, such as in gametogenesis [48]. *ERK* deficiency leads to abnormal reproductive function, the knockout of *ERK* in mice results in abnormal spindle assembly [38], and the inhibition of *ERK* results in suppressed cell growth of PGCs [49]. In meiosis, *P90rsk* phosphorylates *Erp1* [50,51] and plays a major part in the MAPK pathway [52]. The removal of the P90rsk protein from oocytes results in the inability of *C-Mos* to arrest oocytes [53]. In porcine oocytes, MAPK and *P90rsk* are dephosphorylated almost simultaneously after fertilization. The repression of the MAPK pathway by U0126 (MAPK kinase inhibitor) results in the release of 41.44% of MII-arrested oocytes from arrest [54]. In *Asterina pectinifera*, *P90rsk* is necessary for oocyte arrest and is the sole substrate of MAPK necessary for G1 arrest [55]. However, in mice although the MAPK pathway is associated with oocyte arrest, it is not mediated by *P90rsk* [56]. In the present experiment, interference with the upstream *C-Mos* gene resulted in the differential downregulation of both *ERK* and *P90rsk*, predicting that *C-Mos* has a role in regulating the MAPK pathway and the oocyte cycle in *H. cumingii*.

## 5. Conclusions

The *C-Mos* gene was cloned from the *H. cumingii*. An analysis of *C-Mos* expression in different tissues and gonadal tissues at different ages suggested that *C-Mos* may be involved in the gonadal development of the *H. cumingii*. In situ hybridization results showed that clear hybridization signals were found in the oocytes, oocyte nucleus and oogonium, and a small number of hybridization signals were found in the follicular wall of the male gonads. The results of RNAi experiments suggested that *C-Mos* had an association with the MAPK pathway and *P90rsk* in the *H. cumingii*. In conclusion, *C-Mos* is involved in the regulation of gonadal development in *H. cumingii*, but the exact process needs to be further investigated.

## Figures and Tables

**Table 1:** Primers used in this study.

Primer Name	Sequence (5′ to 3′)	Application
M-OUTER	CACCATGTCTAAATCCGGCCCTGTCT	3′RACE
M-INNER	CAGAAGTTGAACGCAGGGCCAGATTT	3′RACE
qPCR-Mos-F	AGTATGAACGAACATCGGAGG	qRT-PCR
qPCR-Mos-R	TGAGAACATCCAAAGTCGCC	qRT-PCR
qPCR-P90rsk-F	GGTCCTATGGCGTGCTAATG	qRT-PCR
qPCR-P90rsk-R	GATTCTTCCAGTCTATGCTTGC	qRT-PCR
qPCR-ERK -F	AGAATCACGGTAGAAGAGGCT	qRT-PCR
qPCR-ERK-R	TTCCTGTCCATTGGTTGTGT	qRT-PCR
qPCR-EFl-αF	GGAACTTCCCAGGCAGACTGTGC	qRT-PCR
qPCR-EFl-αR	TCAAAACGGGCCGCAGAGAAT	qRT-PCR
ISH-MOS-F	TACATACGCCTACCGTGCCC	ISH
T7 + ISH-MOS-R	TAATACGACTCACTATAGGGGTCCCATACGCAACAACCC	ISH
T7 + RNAi-MOS-F1	TAATACGACTCACTATAGGGAGGCTGTGTTTTTGCTCTGA	RNAi
T7 + RNAi-MOS-R1	TAATACGACTCACTATAGGGGCCGCTGGATTTTTCGTAA	RNAi
T7 + RNAi-MOS-F2	TAATACGACTCACTATAGGGACTTGGCGACTTTGGATGT	RNAi
T7 + RNAi-MOS-R2	TAATACGACTCACTATAGGGCGTTTCAGTTACCGTAGGCA	RNAi
T7 + RNAi-MOS-F3	TAATACGACTCACTATAGGGACTGTGAGTCCCACTGAAAGAT	RNAi
T7 + RNAi-MOS-R3	TAATACGACTCACTATAGGGACTAAGACAGCCAAGGTTTCC	RNAi

## Data Availability

All experimental data for this article can be obtained by contacting the corresponding authors.

## Supplementary Materials

The following supporting information can be downloaded at: https://www.mdpi.com/article/10.3390/biom13060931/s1, Table S1: List of amino acid sequences used for multiple alignment analysis and NJ phylogenetic tree of C-Mos protein in *H. cumingii*.
