# Supplementary material for: Identification and Characterization of C-Mos in Pearl Mussel Hyriopsis cumingii and Its Role in Gonadal Development
Source: Biomolecules. 2023 Jun 1;13(6):931. doi: 10.3390/biom13060931 (PMC10296756; doi:10.3390/biom13060931)
Supplement: Supplementary file 1 [file biomolecules-13-00931-s001.zip › biomolecules-2421599-supplementary.pdf]

Identification and Characterization of *C-Mos* in Pearl Mussel *Hyriopsis cumingii* and Its Role in Gonadal Development

Supplementary Table S1. List of amino acid sequences used for multiple alignment analysis and NJ phylogenetic tree of C-Mos protein in *H. cumingii*.

Accession Numbers	Species	Amino acid sequences
XP_052244251.1	Dreissena polymorpha	MLERQSSRGNLHRQSTLRNINKHSCLQRQKSRHTLQRQHSN VVLQAKISDVNNNINVNSVLSVTHVSPRVRHLLLKHTKFGFI DRFQNLSLAGSSADIKSTESLPSILTESDTLIRDCQMPESLFRD DFTASKTINKDDFELGRVLGAGGFGSVYLGIFQKQKVAIKV MHKYTKNPAAQIESFKAELHVMRFNHPNIVRTLAATHIDKF DEGAWIIMEYVGFSNLYKLLSDSEEVIANERRLKFAIQIASAL AYAHKHKVAHLDLKPANILITDDDNCKVGDFGCSQKVEID TGVVSPTNRSILTGTFAYRAPELLRGEPPTFKADVYSFGITLW QLKSRETPFCNQNQHVVIFAVVANGMRPPDMSPAETDPFEL SYKDLYAQCWTANPLDRPSASEVVDVLNIWKLHL
XP_021343629.1	Mizuhopecten yessoensis	MVMVKVREETHSFNHGHQHTPVHGHRHVHHHHGHNSPV HTLKSLFGKYRHLRCSSPKLCLSTPKSKRLSESKRNCLGGEIR RPFLINKKGGEDNDSVFIKEDFKLGKLLGAGGFGSVYMANG KSGHGSMAIKVLKKPTMSKNPDAMFESFKAELRCMQLSHP NVVQSLGATHMTSFEEGAWVVMEYVGERTLQAVLNESQV DITIELRLKFAIQIAQGLKYLHDNSIVHLDIKPANILITPDGDC KIGDLGCSQILEEGTGRVSPTQRSSLTGTFAYRAPELLRGDAP TRKADIYSFGITMWQMLARDNPYGNENQHVVIFGVVAYGH RPKHPNMSEQNPFELCYRDLYSQCWDSTPQNRPSADELVEV LDIWKNQL
XP_050403259.1	Patella vulgata	MPADVNKVSEILMVSESCFVLDDFEVLPQTPFEDLASRFLNK DDYDLGKLLGAGGFGSVYLASYKNKITAVKTLHKVTKNPK AQMESYKAELNVYNWKHPNIVRTVAATSIEQYDTGAWIVM EYIGSRNLQMLINDTEEELDQQRRLKYSLEVASALEFTHSNHI VHLDLKPANILLTERDSCKLGDFGCCQRVEEDTGRVSPTNRS ALTGTFAYRAPELLRGGAPTLVADVYSYGVTLWQLLSRETPY SNQNQHVVIFSVVSKHLRPPHPDVGDDPFELLYQELYTQCW SANPETRPTSRDLVELLKLWKYYM
XP_011422084.2	Crassostrea gigas	MKIASYIQSHQMTKTSFTTRSWQKLKHLLSKYKRSRGVIRKV MPKISFANNKKLKLVSKDDVKLGRLLGAGGFGSVYYGSYRQ RDVAVKIMHKQSKNPEAQIESFKAELHVLDFEHPNIVKTLA ATPFEEFKEGAWIVMEYAGSRTLQSMINNEELCQETRIRFAI QMSDALHYIHDNHVLHLDLKPANILITARGDVKMADFGCS QKVELDTGLVSPTQRSLLTGTFAYRAPELLKGQVPSNKADIY ALGVTLWQMLARENPYGNENQHVVIFSVVAYGHRPPHPEI DLDPFEECYRDLYTQCWSATQFDRPSAKELHETLKIWKKHM

CAC5414491.1	Mytilus coruscus	MQIKNEHNLCSVVKGKKYSLSSLKGINQCQHILRKYKLFRK<br>KLGQFAKRIVMPVLAQNENQRDEFKRPHAIVCTRHRNASFR<br>KEEVVLGRLLGTGGFGSVYRGKYKDSTVAVKVMHRVTKNP<br>AAQLESFKAELNTIGFDHENIVKTITATSLETFDQGAWIVME<br>YAGRRTLQSLVDDDSVSLGPRRRVKFSQQIAEALKYAHDMK<br>IVHLDLKPANILITPGGRCKVADFGCSQKVEIDTGIFSPTQRSI<br>LTGTFAYRAPELLKGEAPSKKADIYALAVIMWQLLSRQTPFS<br>NENQHVVIFGVVAYGQRPKHPEIEVDPFEESYRDLYSQCWL<br>PCPLDRPTAGEIAELLNIWRGYM
XP_033738057.1	Pecten maximus	MFRLTSVDLYMRVHKVLNLRTIKMVMVKVREETHTFHHGH<br>HHHTPTRGHLVHHRHGHNSPAHTLKSLFGKYRHLRCSSPK<br>PNISTPKSKRLSESKRNCLSSEFRKPFLIKKNGAADDDDSVFIK<br>EDFKLGKLLGAGGFGSVYMANGKSGHDSIAIKVMKKPTMS<br>KNPDAMFESFKAELRCMQLAHPNIVQSLGATHMTSFEDGA<br>WVVMEYAGDRTLQTALNESHVDLTIEIRVKFSTQIAQGLKYL<br>HDNNIVHLDIKPANILITPDGVCKIGDLGCSQILEEGTGKVSP<br>TQRSSLTGTFAYRAPELLRGDAPTRKADIYSYGVTMWQMLA<br>RDNPYGNENQHVVIFGVVAYGHRPKHPNMSEQCPFELCYR<br>DLYSQCWDSVPENRPSADELVKVLDVWKKNV
XP_046364430.1	Haliotis rufescens	MPKVAEDADDGPTEDGTDIAMVCDVIYRQSRSLSLPNNTYD<br>YTIESLSGATLSKKDVSLGRLLGSGGFGSVYLGTYKNKEVAV<br>KILHAVTKNPQAQIQSFKAELAILNFKHPHIIRTITATTLEDF<br>NDGAWVVMEYVSDQTLHGVINNMDQQLCMVRRLKYALQI<br>ASAVQYAHDNCIVHLDIKPVNILLTQDDDCKLGDFGCCQE<br>VEFNTGRVSPTNRSALTGTFAYRAPELLKGGPPSLQADIYSFG<br>VTLWQMLSRETPYANENQHVVIFGVVAYGLRPKHPVIGDEP<br>FDKLYQDLYTQCWVAGPEDRPTSTDLMEILETWKQYLD
XP_045157467.1	Mercenaria mercenaria	MLERQHSIGQTSPKHTPNRNHINRHSCLQRQKSRRLFQKQR<br>TSVCLFSSKPADDTLNNNGEKVVSAKLRRMLLKHTKFGFIES<br>FQNLTVKQVCDTDCRVKGESDQLVQNYPMPDNLVFDSQIS<br>AKIINKEEFELGRVLGAGGFGSVYLGSFKRQTVAIKVMHKYT<br>KNPAAQLESFRAELHVMRFNHPNIVKTLAATHIDQFDDGA<br>WVVMEYIGKSSLQGIINDHSEPFDSDRRLKYSIQVASALAYA<br>HKNKVAHLDLKPANILITSTDDCKVGDFGCSQRVEFDTGIVS<br>PTNRSILTGTFAYRAPELLRGEPPTFKADVYSYGITMWQMKS<br>RETPFTNQNQHVVIFGVVANGLRPKDPEPSETDPFELSYKDL<br>YSQCWNACPLDRPSAQELVELLNIWKENL
XP_031759862.1	Xenopus tropicalis	MPSPIPVERFLPRDLSPSIDLRPCSSPLELSHRKVPGSGRHRLL<br>PPRLAWCSIDWEQVRLLEPVGSGGFGSVYRAIYKGETVALKK<br>VKRCTKNSLASRQSFWAELNAARLRHPHVVRVLAASASCP<br>GDPGCPGTIIMEYAGTDTLHGRIYGRCPPLGAAVCMRYARH<br>VADGLCFLHRDGVVHLDLKPANVLLAPGGLCKIGDFGCSQ<br>RLRDGDSAGGEPCCTQLRHVGGTYTHRAPELLKGEPVTAK<br>ADIYSFAITLWQMVSRELPYTGDRQCVLYAVVAYALRPEMG<br>PLFSCTEEGRAVRHIVQSCWAARPEERPSAEQLLERLEQESA<br>LYRGVSSSPSAQYTSPPLSAAHRGP

NP_005363.1	Homo sapiens	MPSPLALRPYLRSEFSPSVDARPCSSPSELPAKLLLGATLPRAP<br>RLPRRLAWCSIDWEQVCLLQRLGAGGFGSVYKATYRGVPV<br>AIKQVNKCTKNRLASRRSFWAELNVARLRHDNIVRVVAAS<br>TRTPAGSNSLGTIIMEFGGNVTLHQVIYGAAGHPEGDAGEP<br>HCRTGGQLSLGKCLKYSLDVVNGLLFLHSQSIVHLDLKPANI<br>LISEQDVCKISDFGCSEKLEDLLCFQTPSYPLGGTYTHRAPEL<br>LKGEGVTPKADIYSFAITLWQMTTKQAPYSGERQHILYAVV<br>AYDLRPSLSAAVFEDSLPGQRLGDVIQRCWRPSAAQRPSARL<br>LLVDLTSLKAELG
NP_064405.2	Mus musculus	MPSPLSLCRYLPRELSPSVDSRSCSIPLVAPRKAGKLFLGTTPP<br>RAPGLPRRLAWFSIDWEQVCLMHRLGSGGFGSVYKATYHG<br>VPVAIKQVNKCTKDLRASQRSFWAELNIARLRHDNIVRVVA<br>ASTRTPEDSNSLGTIIMEFGGNVTLHQVIYGATRSPEPLSCRE<br>QLSLGKCLKYSLDVVNGLLFLHSQSILHLDLKPANILISEQDV<br>CKISDFGCSQKLQDLRCRQASPHHIGGTYTHQAPEILKGEIA<br>TPKADIYSFGITLWQMTTREVPYSGEPQYVQYAVVAYNLRPS<br>LAGAVFTASLTGKTLQNIIQSCWEARALQRPGAELLQRDLK<br>AFRGALG
NP_991143.1	Danio rerio	MPSPIPVTRLLPKDFGLEFGACSSPLTKTASGSTLRVPTNKFH<br>GKVAHRLWSSVIHWRELQALEPIGSGGFGTVFRGTYFGETV<br>AVKKVKCVKNKLASRQSFWAELNAAHLHHQNIVRVLAAT<br>TCTPAHLNTKDNIGTIVMEFAGNINLQKLIYGLTDLLPVEKC<br>IKYSIDIARALQHLHAHGVVHLDLKPANVLLSEQGVCKIAD<br>FGCSFKISSTSDTVTHMNEIGGTFTHRAPELLKGEEVSPRVDV<br>YSFGITLWQLLTREPPYEGDRQYILYAVVGYNLRPLTSRNVFT<br>QFFIGQNCQKLISRCWDGDPSIRPTADKFVDELSVLL
XP_022319224.1	Crassostrea virginica	MKTTSYSYSHQMTKTTFTTRSWQKLKHLLSKYKRSRGGFFRK<br>VMPKISLVHNKKLTHVNKEEVKLGRLLGAGGFGSVYLGSYR<br>QHDVAVKIMHKQSKNAAAQMESFKAELHVLDFEHPNIVRT<br>LAATPFEEFPEGGWIVMEYAGSRTLQSMLNSEELCQETRIQF<br>ALQMSDALQYIHQNHVLHLDLKPANILITTRGDIKIADFGCS<br>QKVEVDTGLVSPTQRSLLTGTFAYRAPELLKGQVPSQKADIY<br>ALGVTLWQMLARENPYGNENQHVVIFSVVAYGQRPPHPQI<br>EMDPFEECYRDLYSQCWAVSQLDRPSAKELHETLKIWKKYM
CAG2253472.1	Mytilus edulis	MQIKSKQNLCSVVKDKKYSSSSLKGLNQCQHILRKYKLFRKK<br>LGQFAKRIVMPVLAHKEKKRDEFKRPHAIVSSRHRNTSLKK<br>EEVVLGRLLGTGGFGSVYRGKYKDSTVAVKVMHRVTKNPA<br>AQLESFKAELNTIGFDHENIVKTITATSLDAFDQGAWIVMEY<br>AGHRTLQSLIDDDSISLGPCRRVKFSQQIAEALKYAHDMKIV<br>HLDLKPANILITPDGRCKVADFGCSQKVEIDTGIVSPTQRSIL<br>TGTFAYRAPELLKGEAPSKRADIYALAVIMWQLLSRQTPFSN<br>ENQHVVIFGVVAYGQRPKHPEIDVDPFEESYRDLYSQCWLP<br>CALDRPTAGEIAELLNIWRGQGFGIATIYMERNTVFKQNVW<br>NFMYGKNGSSEHSDSEGEITPPIQAEKTVREVKTYSVLPPPAD<br>YTPTEPLEDNKGEDEPSDSSSSSDSDKPARKRKRKNRRHTEK<br>CKEEKSHQHDTSDKKLTKNQKRKLKKKRRKEKQKNEDKSV<br>TFSFISSEDNQISAETTSNNEIQDRVADLSNFFDAVWDVYKL<br>QEREKSDEQQDVFDELNKSLSLLDDHSKEIESQLNIIHNIKRLI<br>LLGDMKSAGDLMSSMKSDGCFMSEGVNHLIIRLFEYWMKDI<br>SDKG

>XP_046567047.1	Haliotis rubra	MPKFEEHGADDGQTDDVADIEMVCDAIYRQSRSLSLPNNTF<br>DHTIESLSGETLSKKDVSLGRLLGSGGFGSVYLGTYKNNEVA<br>VKIFHAVSKNPLAQVQSFKAELAILNFKHPHIIRTLTATTLDD<br>FNDGPWVVMEYVSDQTLQSVINDMDQQLCMVRRLKHALQ<br>IASAVQYAHDNCIVHLDIKPVNVLLTQDDDCKLGDFGCCQ<br>EVEFNTGRVSPTNRSALTGTFAYRAPELLKGGAPSLQADIYS<br>YGVTLWQMLSRETPYANENQHVVIFGVVAYGLRPKHPVIG<br>DEPFDKLYQDLYTQCWVAEPDDRPTSSELMEILETWKQYID
